# Comparative study of nonlinear properties of EEG signals of normal persons and epileptic patients

**DOI:** 10.1186/1753-4631-3-6

**Published:** 2009-07-20

**Authors:** Md Nurujjaman, Ramesh Narayanan, AN Sekar Iyengar

**Affiliations:** 1 Plasma Physics Division, Saha Institute of Nuclear Physics, 1/AF, Bidhannagar, Kolkata – 700064, India; 2 Current address: Laboratorio Associado de Plasma, Instituto Nacional de Pesquisas Espaciais, Av. dos Astronautas, 1758 – Jardim da Granja 12227-010 Sao Jose dos Campos, SP, Brazil

## Abstract

**Background:**

Investigation of the functioning of the brain in living systems has been a major effort amongst scientists and medical practitioners. Amongst the various disorder of the brain, epilepsy has drawn the most attention because this disorder can affect the quality of life of a person. In this paper we have reinvestigated the EEGs for normal and epileptic patients using surrogate analysis, probability distribution function and Hurst exponent.

**Results:**

Using random shuffled surrogate analysis, we have obtained some of the nonlinear features that was obtained by Andrzejak *et al*. [Phys Rev E 2001, 64:061907], for the epileptic patients during seizure. Probability distribution function shows that the activity of an epileptic brain is nongaussian in nature. Hurst exponent has been shown to be useful to characterize a normal and an epileptic brain and it shows that the epileptic brain is long term anticorrelated whereas, the normal brain is more or less stochastic. Among all the techniques, used here, Hurst exponent is found very useful for characterization different cases.

**Conclusion:**

In this article, differences in characteristics for normal subjects with eyes open and closed, epileptic subjects during seizure and seizure free intervals have been shown mainly using Hurst exponent. The H shows that the brain activity of a normal man is uncorrelated in nature whereas, epileptic brain activity shows long range anticorrelation.

## Background

The brain is a highly complex and vital organ of a human body whose neurons interact with the local as well as the remote ones in a very complicated way [1-4]. These interactions evolve as the spatio-temporal electro magnetic field of the brain, and are recorded as Electroencephalogram (EEG) [1,4-6]. Though the detail link between EEGs and the underlying physiology is not well understood, the former is widely used for detection and prediction of epilepsy, localization of epileptic zone and characterization of the pre and post-ictal [1,6,7] using linear and nonlinear analysis techniques [1,6-11]. Though mainly nonlinear methods have been applied to predict the onset of epileptic seizure and localizing epileptic regions, limited progress has been achieved so far [11]. Even some negative results have also been reported like linear measures are better than nonlinear measures [12,13], seizure is not a low dimensional process [14], it lacks determinism [8,15,16], etc. Hence finding proper analysis techniques is also one of the main issues and experts try out different analysis tools for characterizing the normal and diseased brain states, especially the epileptic brain.

In 2001, Ralph G. Andrzejak, *et al*. and later some other authors [17,18] have analyzed five sets of EEG signals [19] each set containing 100 epochs to study the determinism in the brain dynamics for five different physiological and pathological conditions. Sets A and B are for normal persons with eyes open and closed respectively and recorded extracranially. Sets C and D were recorded intracranially from the hippocampal formation which was nonepiletogenic of the opposite hemisphere of the brain and from within the epileptogenic zone of an epileptic patient during seizure free intervals respectively. Set E was recorded intracranially from the epileptic zone during seizure. The details of the experiments and the conditions have been described in Ref [1]. R.G. Andrzejak, *et al*. [1] had shown that the normal healthy subject with eyes closed and open shows stochastic behavior using amplitude adjusted Fourier transform surrogate analysis where discriminating statistics were the effective correlation dimension and nonlinear prediction error whereas, using delay vector variance discriminating statistics, significant nonlinear determinism was shown in the same subject [17]. So two conflicting results were obtained for the same subject using nonlinear methods. In the case of epileptic patients during seizure and seizure free intervals, determinism was shown using two different methods [1,17] though other studies show lack of determinism for different epileptic patients during seizure [12,15,16,20].

On the other hand, characterization of EEGs by scaling properties of the signal is also a major area of research interest [8-10,21-27]. Power spectral exponent has been used to characterize the different subjects with different physiological conditions [8,9,24,25] and the same exponent has also been used to estimate the correlation dimension (*D*_*corr*_) [8]. Fractal dimension and hurst exponent have also been used to characterize the EEGs [26,27]. Hence a number of experts prefer scaling properties to characterize EEG for different physiological and pathological conditions [8].

In this paper, we have reinvestigated the EEG data studied in Refs. [1,17,18] by random shuffled surrogate analysis using *D*_*corr *_as discriminating statistics in order to find determinism in the signal [28-30] and the results have been compared with earlier analyses [1,17]. Probability distribution function shows a difference between normal and epileptic brain states and this has been discussed in latter Section. Finally, we have quantified the five different physiological brain states by Hurst exponent (H) which has been estimated using *R*/*S *analysis [31].

## Results and discussion

### Surrogate analysis

Surrogate analysis determines the dynamics in the time series: whether it is governed by stochastic or deterministic process [28-30].

**Figure 1:**
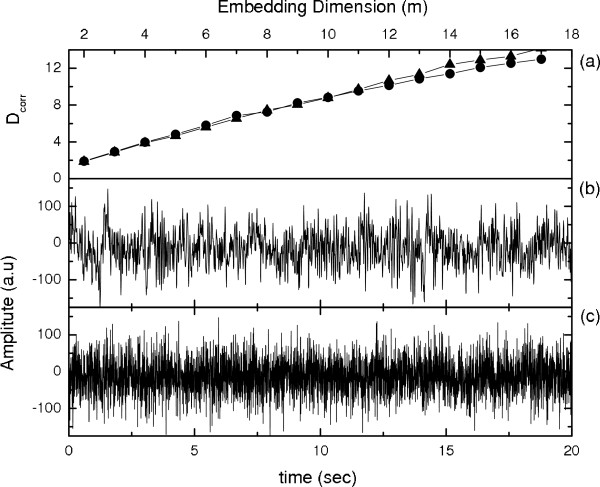
**Surrogate analysis for data Set A: **(a) Variation of *D*_*corr *_with m. Black dot for (b) Original data; and triangle for (c) Surrogate data.

**Figure 2:**
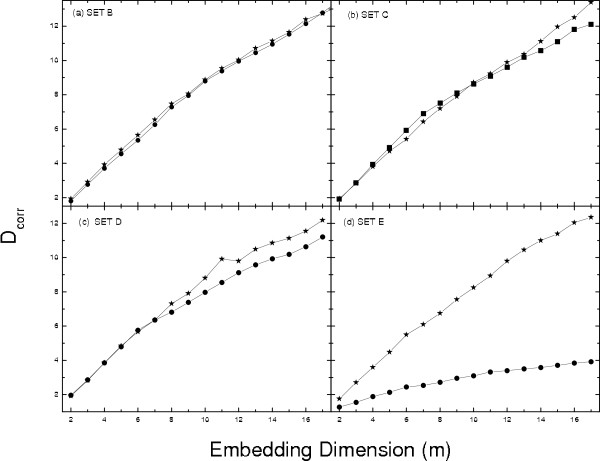
**Correlation dimension: (black dot with a dash either side for original data) and (black star with a dash either side for surrogate data) for data Set B, C, D and E**.

The surrogate data has been generated by Random Shuffled (RS) surrogate method, in which the signals were shuffled randomly so that the probability distribution is same but the temporal correlations are destroyed [28,29,32]. *D*_*corr *_which gives us a measure of the complexity has been estimated for both the original and the surrogate data of the data sets A, B, C, D, and E respectively. Fig 1(a) shows that the *D*_*corr *_increases with the same trend for both the original [Fig 1(b)] and the surrogate data [Fig 1(c)] for the normal persons with eyes open [Set A]. A similar trend is observed in the case of the persons with eyes closed, i.e., for Set B [Fig 2(a)]. This shows that the brain activity of a normal person is stochastic in nature agreeing with the analysis by Andrzejak, *et al*. [1]. For an epileptic patient we found a different behavior during seizure free intervals and during seizure activity. The EEG signals, at seizure free state, recorded both from the hippocampal formation of the opposite hemisphere of the brain [Fig 2(b) Set C] and within the epileptogenic zone [Fig 2(c) Set D] show almost same trend in the increase in *D*_*corr *_for the surrogate data as well as for original data, except for a small separation at higher embedding dimensions, which may be due to a high dimensionality of the system. During seizure activity [Fig 2(d) Set E], *D*_*corr *_saturates with embedding dimension indicating low dimensional deterministic dynamics and these results agrees well with previous analyses [1,17]. For sets A-D, as there is no saturation in *D*_*corr *_at higher embedding dimension [Fig 1(a) and Figs 2(b)–(d)] and hence it is difficult to estimate actual *D*_*corr*_. In Ref [1]*D*_*corr *_was computed based on quasiscaling regions, but such an estimation is very much dependent on the variations of the time-frequency-energy characteristics rather than any nonlinear dynamics. Hence this may be inadequate to characterize epilepsy or diseased brain states for clinical application [33].

### Probability distribution functions

**Figure 3:**
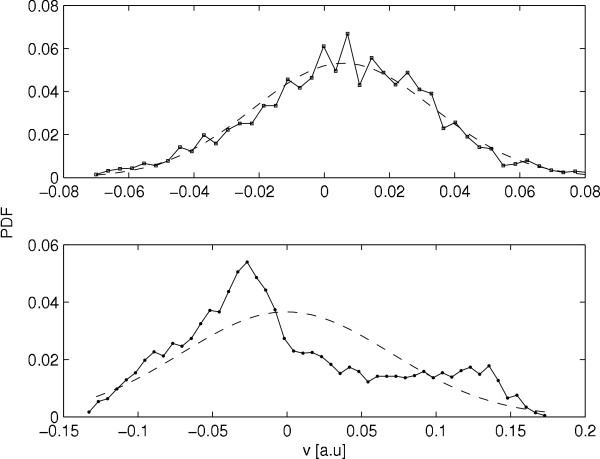
**Typical PDF for normal person with eyes closed (up) and for epileptic patient during seizure (bottom)**.

As we have observed from the surrogate analysis that nonlinear dynamics is responsible for epileptic patients during seizure, we have compared the probability distribution function (PDF) of a normal case and an epileptic person during seizure. The PDF for sets A and E have been shown in Figs 3(a) and 3(b) respectively. Fig 3(a) shows that for a normal healthy person with eyes open, the PDF is Gaussian in nature, whereas for epileptic patients during seizure, it is nongaussian [Fig 3(b)] signifying an intermittent nonlinear effect. But for other three cases this feature is not so clear. So we feel that the PDF may also be useful to differentiate a brain activity of an epileptic patient during seizure from other state.

### Hurst exponent

Since one of the major emphasis of epilepsy investigation is to predict their occurrence, it is necessary to know how the data is correlated. We have carried out a study of the Hurst exponent (H) which has been estimated using Rescaled range analysis (*R*/*S*). This method was proposed by Hurst and well established by Mandelbrot, and Wallis [31]. For a given set of data series, *R*/*S *is defined as [31,34]:(1)

**Figure 4:**
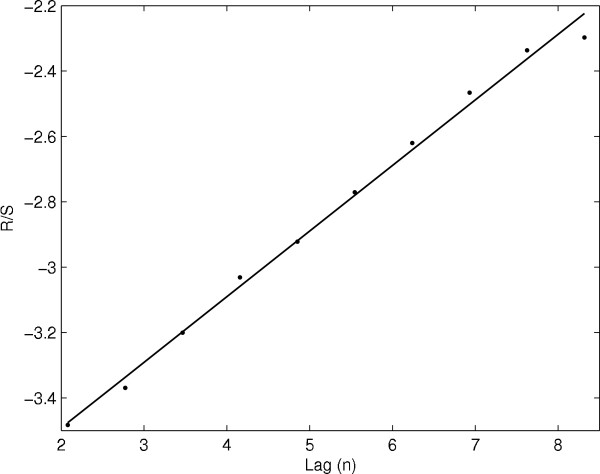
**Typical R/S vs lag n plot**.

Here , where , *S*^2^(*n*), and n are respectively the mean, variance, and time lag of the signal. The expected value of the *R*/*S *scales like *cn*^*H *^as *n *→ ∞, where H is called the Hurst exponent, and can be estimated from the slope of typical plot  vs lag (n). For a given signal, we divided the data into nonoverlapping blocks of equal length and *R*/*S *has been calculated using the Equation 1 and the average value of *R*/*S *has been plotted as a function of lag in a log - log plot as shown in Fig 4 and estimated H from the slope of the curve. For random data *H *= 0.5, while *H *> 0.5 for the data with long range correlations, and *H *< 0.5 indicates the presence of long-range anticorrelation or antipersistency in the data.

**Figure 5:**
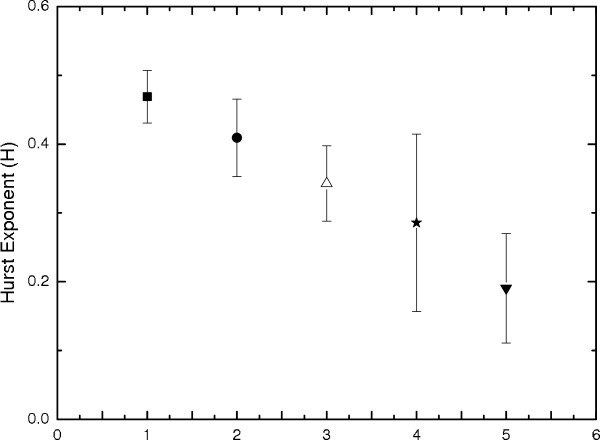
**Average H with standard deviation error bar, Hurst exponent for hundred time series and average H are represented by: square for normal men with eyes open [set A]; black dot for normal men with eyes closed [set B]; triangle and star are for the epileptic patients during seizure free interval from two different locations [set C and D]; upside down triangle for the epileptic patients during seizure [set E]**.

**Figure 6:**
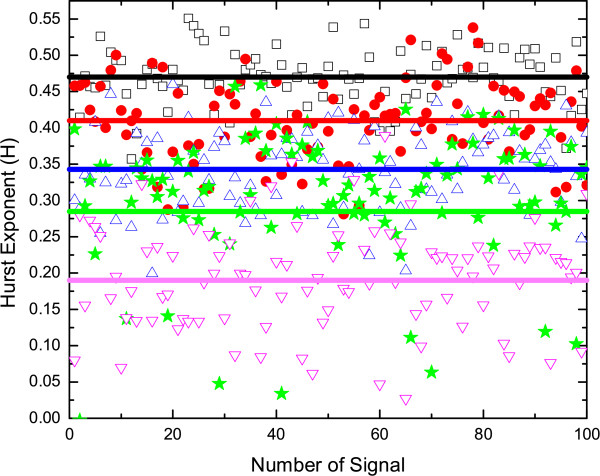
**Hurst exponent for hundred epochs and average H are represented by: black square and black line for set A; red dot and red line for set B; blue triangle and blue line for set C; green star and green line for set D; pink upside down triangle and pink line for set E**.

The estimated average Hurst exponent (<*H *>) with an error bar of 100 epochs for all the five EEG data sets (viz. A-E) have been shown in Fig 5. The solid box and black dot show the <*H *> for sets A and B respectively. For a normal person with eyes open (set A), the average H (<*H *>) ≈ 0.47 whereas, for the data set B, i.e., for a normal person with his eyes closed, <*H *> ≈ 0.41. The <*H *> ≈ 0.5 for a normal man with eyes open indicates that the signals are uncorrelated over long time scales signifying stochasticity of the normal brain. But with eyes closed state, decreases in H (<*H *> ≈ 0.41) may be due to the imposition of some extra constraint, which may influence the system towards an antipersistent state. The <*H *> for epileptic patients are shown by up triangle for set C; star for set D; and down triangle for set E respectively. <*H *> ≈ 0.34 and 0.29 for the EEGs recorded at the hippocampal formation and epileptic zone for the seizure free intervals of epileptic patients respectively and during seizure (data set E), we get the lowest H (<*H *> ≈ 0.19). The H for epileptic patient during seizure and seizure free intervals show anticorrelation which may be due to epileptiform discharges during seizure free intervals indicating that a large discharge is always followed by a small one. The physiology behind the epileptiform discharge is due to the chronic dysfunction or "defect" in the epileptic brain, i.e., the epileptic brain is not normal even during seizure free time [35]. Though the hippocampus was nonepileptogenic for these subjects, its H is still less than a normal person which may be due to its participation in secondary, nonautonomous epileptic processes initiated by the epileptic zone [1]. The wide dispersion in H for the EEG recorded from the epileptic zone in seizure free intervals (star) indicates that the epileptiform discharges are intermittent probably due to the chronic presence of abnormal epileptogenic tissues [36,37]. EEG recording during seizure may not be economical and hence it may be better to locate epileptic zone by recording EEG during seizure free intervals. These analyses show the possibility of detecting the onset of the seizure state from the time dependent Hurst exponent estimated during the transition from normal to seizure state [35]. Fig 6 shows the Hurst exponents for all the epochs of five different sets that have been discussed above.

## Conclusion

In this paper we have reinvestigated the EEG data of normal and epileptic subjects to get an insight into the brain dynamics at different imposed and diseased conditions using RS surrogate analysis, PDF and H exponents. From these analysis we have found that RS and PDF may be useful to find a broad difference between normal and epileptic subjects but not helpful for constrained and seizure free intervals. Whereas, using H exponent, we have obtained differences in characteristics for normal subjects with eyes open and closed, and epileptic subjects during seizure and seizure free interval. The H shows that the brain activity of a normal man is uncorrelated in nature whereas, epileptic brains show long range anticorrelation.

## Competing interests

The authors declare that they have no competing interests.

## Authors' contributions

MN and RN had carried out the time series analysis. MN, RN and ANSI prepared the manuscript. All the authors read and approved the final manuscript.
